# Supplementary material for: Reward biases spontaneous neural reactivation during sleep
Source: Nat Commun. 2021 Jul 6;12:4162. doi: 10.1038/s41467-021-24357-5 (PMC8260738; doi:10.1038/s41467-021-24357-5)
Supplement: Supplementary file 1 — Supplementary Information [file 41467_2021_24357_MOESM1_ESM.pdf]

Supplementary Information

Supplementary Figures

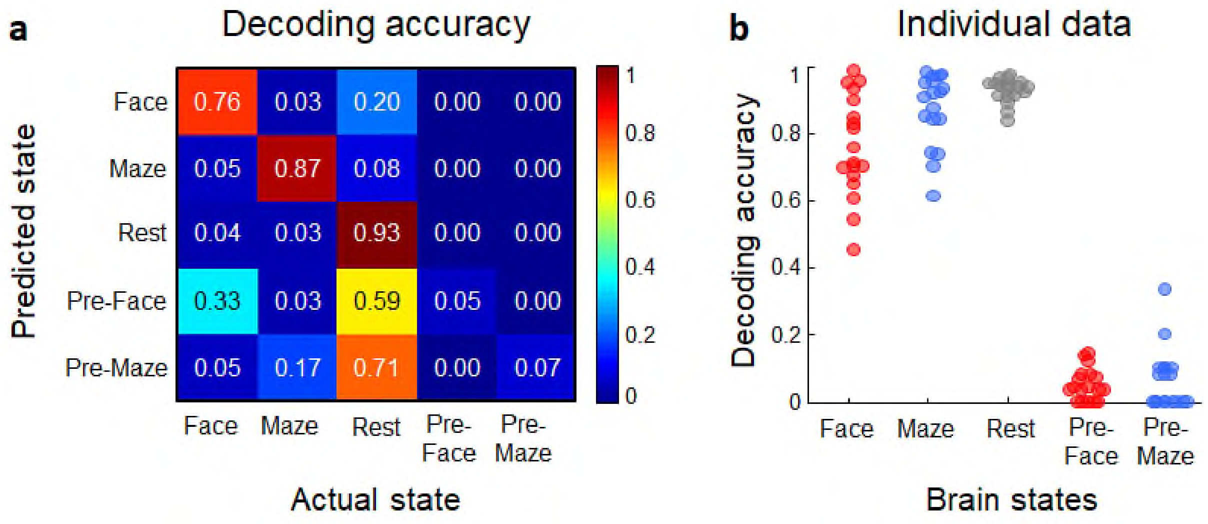

<u>Supplementary Figure 1.</u> <u>Task-related decoding accuracy.</u> **<u>(A)</u>** Confusion matrix for the 5 states classified using the fMRI data of game session. Decoding accuracy resulted from a leave-one-out procedure and indicated high classification accuracy for the Face, Maze, and Rest states. **(B)** Individual measures of decoding accuracy for predicted vs. actual state (N=18). Source data are provided as a Source Data file.

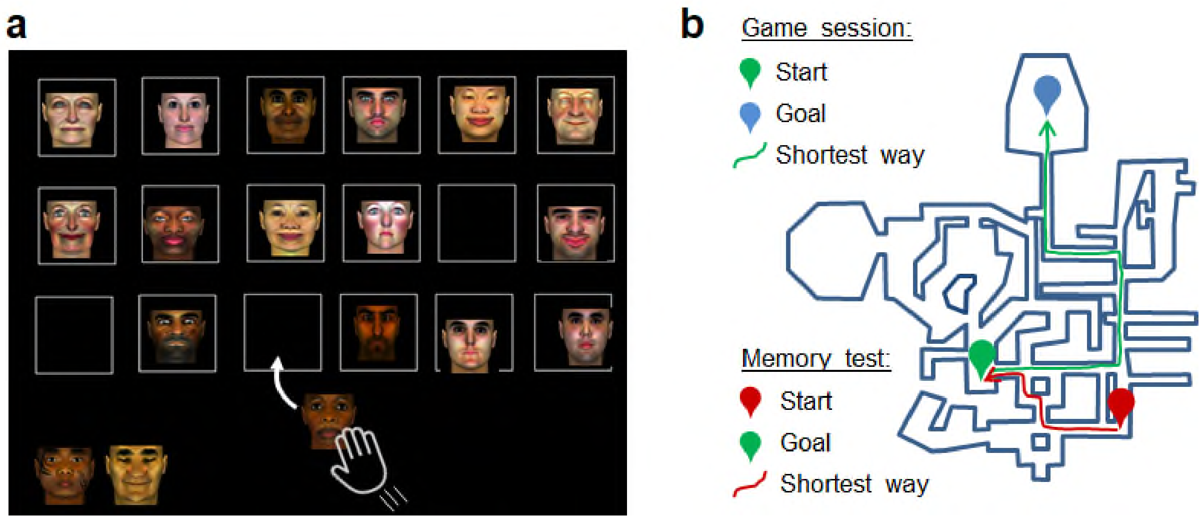

<u>Supplementary Figure 2.</u> Memory tests performed 2 days after the game session. (**A**) Memory test for the face game. Participants had to place each individual face at its correct location (i.e. its location during the game session) within a 3 by 6 grid. (**B**) Map of the maze game with the start and goal locations for game session and Memory test.

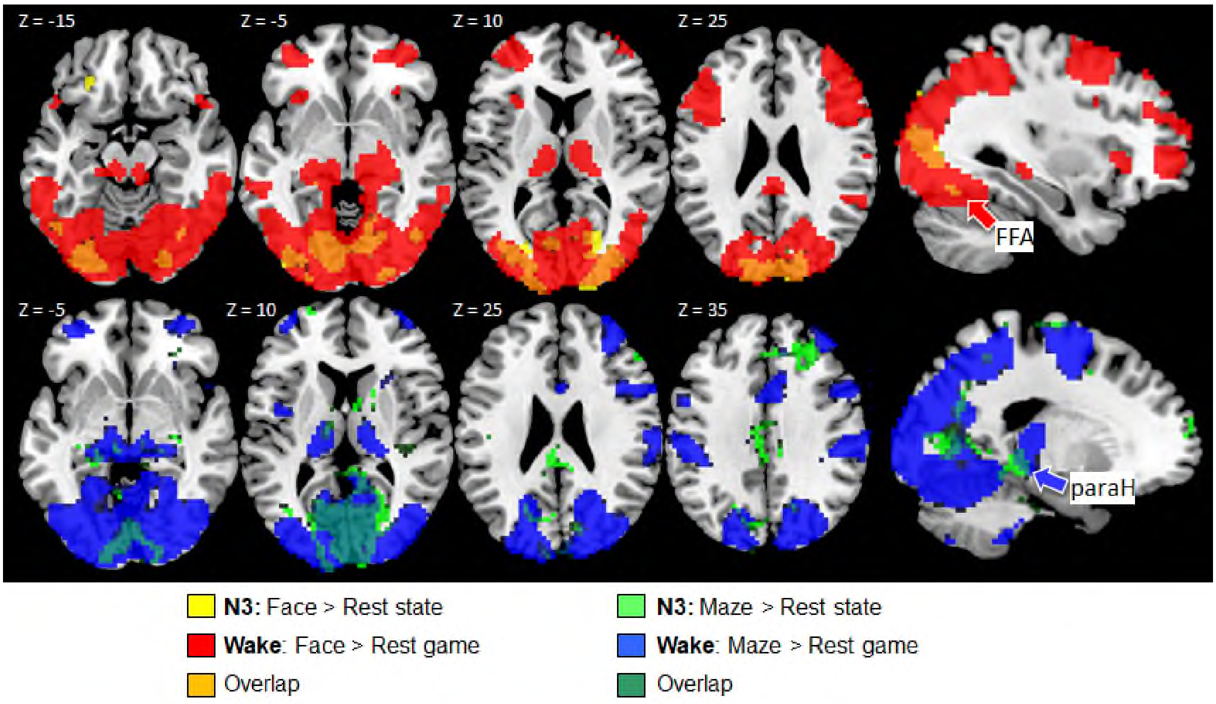

Supplementary figure 3. Game-specific reactivation during N3 (N=13). Upper panel shows the brain regions activated for the Face vs Rest conditions during wakefulness (red) and sleep (yellow), and overlapping activations (orange). Lower panel shows the brain regions activated for the Maze vs Rest conditions during wakefulness (blue) and sleep (light green), and overlapping activations (dark green). N3 indicates N3 sleep stage.

Supplementary Tables

Supplementary Table 1. Main clusters of activation during the game session used as ROIs for the classification (n=18)

Name of the region	Side	X (mm)	Y (mm)	Z (mm)	Z score	P FWE-corr
**Face > maze game**						
Fusiform cortex, encompassing the fusiform face area (FFA)	R	45	-55	-20	4.15	0.012*
	L	-42	-64	-14	5.44	0.004**
Occipital face area (OFA)	R	39	-88	-11	4.50	0.002*
	L	-42	-85	-14	4.05	0.031**
Inferior parietal cortex	L	-54	-61	28	6.34	<0.001**
	R	51	-61	40	5.51	0.005**
Precuneus medial	R	6	-64	37	6.16	<0.001**
Superior temporal sulcus	R	66	-40	-2	5.46	0.001**
	L	-60	-31	-11	7.07	<0.001**
Inferior frontal cortex	L	-51	23	16	6.18	<0.001**
Medial prefrontal cortex	L	0	53	16	4.91	0.012**
**Maze > face game**						
Para/hippocampus, encompassing the parahippocampal place area (PPA)	R	27	-46	-11	7.41	<0.001**
	L	-24	-49	-11	6.79	<0.001**
Precentral gyrus / motor cortex	L	-36	-19	58	7.61	<0.001**
Frontal eye field	R	24	5	55	7.10	<0.001**
	L	-21	-7	67	7.46	<0.001**
Early visual cortex (lingual gyrus)	R	6	-79	-5	7.04	<0.001**
	R	15	-76	-8	6.90	<0.001**
	L	-24	-79	-11	6.66	<0.001**
Precuneus lateral	R	27	-82	31	7.08	<0.001**
	L	-18	-85	31	6.44	0.001**
Cerebellum (vermis)		0	-61	-38	6.21	<0.001**
**Face-rest1 < face-rest3**						
Thalamus	L	-18	-28	-2	6.94	<0.001**
Superior occipital cortex	R	18	-88	31	6.98	<0.001**
	L	-15	-85	31	Inf	<0.001**
Postcentral gyrus	L	-57	-22	25	4.76	0.013**
Precentral gyrus	L	-36	-19	58	4.80	0.003**
Inferior frontal operculum	L	-39	8	25	4.94	0.001**
	R	48	14	25	5.79	0.013**

Putamen	R	24	14	4	4.22	0.035*
Middle temporal lobule	R	36	17	-35	6.49	<0.001**
Supplementary motor area	R	3	11	67	4.48	0.039**
Superior temporal sulcus	L	-42	-31	16	6.07	0.001**
Cerebellum (vermis)	R	0	-61	-38	6.48	<0.001**
Early visual cortex (calcarine)	L	-15	-73	13	4.99	0.035**
	R	6	-76	16	5.79	<0.001**
Medial superior frontal gyrus	R	6	53	13	3.94	0.042*
Rolandic operculum	R	48	-22	7	4.92	0.003*
Fusiform gyrus	L	-27	-64	-11	7.02	<0.001**
	R	24	-52	-14	6.75	<0.001**
**Maze-rest1 < maze-rest3**						
Inferior parietal cortex	R	27	-52	49	5.72	0.002**
Superior parietal cortex	L	-24	-49	58	5.68	0.001**
Middle temporal cortex	L	-51	-49	10	4.99	<0.001**
Superior temporal cortex	R	54	-46	19	4.25	<0.001**
Inferior frontal gyrus	R	54	23	-2	4.16	<0.001**
Insula	R	39	26	-5	4.01	<0.001**
Inferior frontal orbital	L	-33	26	-8	3.88	<0.001**
Medial frontal orbital	R	3	35	-11	5.36	0.11**
Anterior cingulate	R	0	38	1	4.71	<0.001**
Cerebellum (posterior lobe)	L	-12	-49	-50	5.62	<0.001**
Cerebellum (dendate)	R	18	-52	-29	5.79	<0.001**
Fusiform gyrus	L	-30	-55	-14	6.23	<0.001**
	R	24	-64	-8	7.10	<0.001**
Lingual gyrus	R	21	-55	-2	6.73	<0.001**
Paracentral lobule	R	15	-40	52	4.37	<0.001**
Hippocampus	L	-21	-28	-5	5.83	<0.001**
	R	21	-28	-2	5.77	<0.001**

**significant after correction on the whole brain *significant at FWE after correction on small volumes from AAL atlas, see Methods.

Supplementary Table 2. Sleep stages for the sleep session in the MRI scanner for the 13 participants who reached N3 sleep stage (SD: Standard Deviation). Source data are provided as a Source Data file.

Wakefulness	N1	N2	N3
**Duration (minutes, mean ± SD)**			
24.9 ± 16.9	11.4 ± 11.2	20.9 ± 10.3	40.8 ± 17.8
**Percentage of total time spent in the MRI (%, mean ± SD)**			
25.5 ± 16.2	11.9 ± 12.4	21.4 ± 15.6	41.2 ± 15.6

Supplementary Table 3. Likelihood of occurrence of each brain state across distinct sleep stages (mean ± SEM (Standard Error of the Mean); N=13). Source data are provided as a Source Data file.

	Wakefulness	N1	N2	N3
Reward state	0.061 ± 0.022	0.099 ± 0.033	0.158 ± 0.026	0.429 ± 0.070
No-Reward state	0.034 ± 0.010	0.117 ± 0.046	0.150 ± 0.043	0.146 ± 0.029
Rest state	0.903 ± 0.030	0.778 ± 0.068	0.683 ± 0.056	0.418 ± 0.062
Pre-R state	0.001 ± 0.000	0.003 ± 0.001	0.005 ± 0.000	0.004 ± 0.001
Pre-NR state	0.001 ± 0.000	0.003 ± 0.001	0.005 ± 0.001	0.003 ± 0.001

Supplementary Table 4. Correlations between time-courses of state likelihood and power spectrum (rho values; mean ± SEM (Standard Error of the Mean); N=13). Source data are provided as a Source Data file.

	Low delta	High delta	Theta	Alpha	Sigma	Beta
Reward state	0.54 ± 0.32	0.45 ± 0.34	0.27 ± 0.39	-0.02 ± 0.32	0.19 ± 0.31	-0.15 ± 0.32
No-Reward state	0.37 ± 0.21	0.33 ± 0.21	0.22 ± 0.28	-0.01 ± 0.25	0.18 ± 0.26	-0.10 ± 0.27
Rest state	-0.52 ± 0.28	-0.44 ± 0.30	-0.27 ± 0.37	0.02 ± 0.32	-0.20 ± 0.32	0.15 ± 0.31
Pre-R state	0.27 ± 0.20	0.23 ± 0.20	0.16 ± 0.25	0.00 ± 0.18	0.10 ± 0.21	-0.04 ± 0.21
Pre-NR state	0.17 ± 0.19	0.16 ± 0.18	0.12 ± 0.15	-0.01 ± 0.16	0.09 ± 0.16	-0.03 ± 0.17

Supplementary Table 5. Brain activations correlating with the time-course of each game-related state's likelihood during N3 sleep (N=13)

**Name of the region**	**Side**	**X (mm)**	**Y (mm)**	**Z (mm)**	**Z score**	**P FWE-corr**
**Conjunction between Maze state vs. Rest state during N3 and Maze game vs. Rest during wake**						
Para/Hippocampus	L	-21	-31	-2	3.76	0.014*
Early visual cortex (calcarine)	R	18	-100	13	5.37	0.002**
	L	-12	-103	10	5.16	0.007**
Early visual cortex (lingual gyrus)	L	0	-76	1	4.85	0.037**
	L	-24	-85	-14	4.75	0.001**
	R	24	-88	-14	4.74	0.001**
Cuneus	L	-12	-88	34	4.45	0.002**
	R	12	-88	34	4.57	0.001**
Precentral gyrus	L	-39	-19	55	4.17	0.011*
Superior Parietal lobule	R	24	-55	61	3.58	0.041*
**Conjunction between Face state vs. Rest state during N3 and Face game vs. Rest during wake**						
Occipital face area (OFA)	L	-42	-73	7	3.56	0.045*
	R	39	-79	1	3.45	0.040*
Fusiform face area (FFA)	L	-36	-85	-14	3.53	0.042*
	R	24	-85	-14	3.70	0.027*
Early visual cortex (lingual gyrus)	L	-24	-85	-11	3.87	0.007**
Early visual cortex (calcarine)	L	-15	-97	-5	5.01	0.016**
Cuneus	L	-12	-94	19	4.84	0.028**
	R	21	-97	22	4.71	0.045**
Precentral	L	-39	-16	55	3.60	0.041*
Superior parietal lobule	R	27	-52	49	3.40	0.049*

**significant after correction on the whole brain *significant at FWE after correction on small volumes from AAL atlas, see Methods.

Supplementary Table 6. Sleep duration derived from the actigraphy data and subjective sleep quality for the 3 nights prior to the scanning phase, the scanning night itself, and the immediately following night (i.e. preceding the Memory test phase). Data are presented for the participants who reached N3 sleep stage. Note that measurements for the night of the Scanning phase started from the moment participants arrived at home (N=13) (SD: Standard Deviation). Source data are provided as a Source Data file.

Night -3	Night -2	Night -1	Night of the experiment	Night +1
**Sleep duration (hours and min ± SD)**				
8.44 ± 1.27	8.04 ± 0.91	8.27 ± 1.05	7.71 ± 1.56	8.12 ± 1.17
**Sleep quality (from 0 poor sleep to 10 excellent sleep ± SD)**				
7.85 ± 1.34	7.77 ± 1.36	7.77 ± 1.36	7.08 ±1.71	7.77 ± 1.64

Supplementary Note

Replication of the results with only 3 states:

We rerun all the analysis without the pre-Face and the pre-Maze because their duration is shorter than the other states and we wanted to be sure that their improve efficiency of the classifier rather than deteriorate it. Thus, the classifier was trained on only 3 states, including Face, Maze and Rest states. The figure below shows the classification of the fMRI data from the game session when training the classifier on the initial 5 states (Supplementary Fig. 4A and B) and on only 3 states (Supplementary Fig. 4C and D). The confusion matrices of the cross-validation (leave-one-out procedure) shared a similar general pattern but, and as expected, the classifier was slightly less efficient with 3 states, i.e. when excluding the pre-Face and pre-Maze conditions, than with the initial 5 states (Supplementary Fig. 4E). Specifically, a repeated-measures ANOVA on the shared 3 states of interest with Method (5 or 3 states) and Game-related State (Face, Maze, Rest) as within-subjects factors revealed a main effect of Method (F(1,17=, 5.9, p=0.026) due to higher decoding accuracy for 5 compared to 3 states, a main effect of Game-related State (F(2,34)=18.9, p<0.001), higher accuracy for Rest compared to Face and Maze states, and no interaction (F(2,34)=3.6, p=0.37).

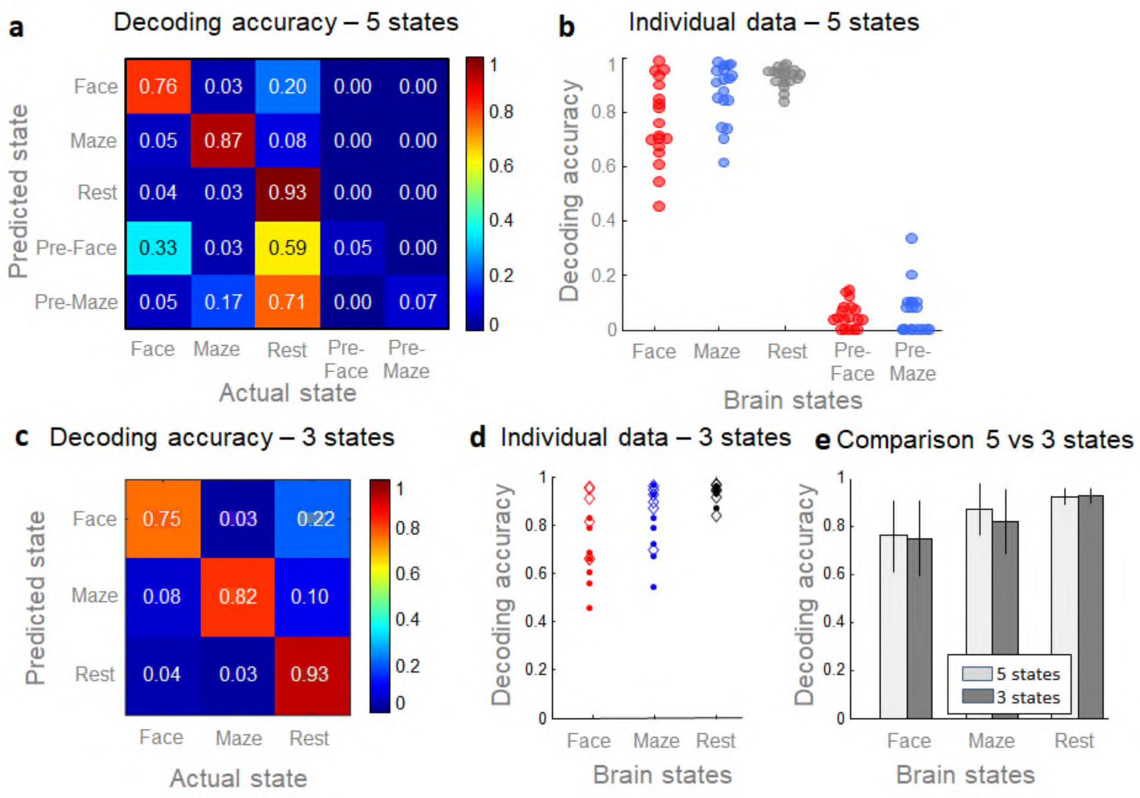

Supplementary Figure 4. Task-related decoding accuracy (N=18). (A) Confusion matrix and (B) individual decoding accuracy measures for 5 states. (C) Confusion matrix and (D) individual decoding

accuracy measures for 3 states. (E) Summary measures for the states Face, Maze, Rest when using 5 and 3 states (mean ± SD (Standard Deviation))

We then performed all the analyses reported in the main manuscript, but now using the output of the classifier on 3 states. For the decoding of the data from the Sleep session, a repeated-measures ANOVA on likelihood measures with Brain state (Reward, No-Reward, Rest) and Sleep stage (wake, N1, N2, N3) as within-subjects factors showed main effects of Brain state ($F_{(2,24)=}$86.3, $P < 0.001$) and an interaction between Brain state and Sleep stage ($F_{(6,72)} = 13.4$, $P < 0.001$) (Supplementary Fig. 5A). Post-hoc planned comparisons first replicated the main results, showing that the Rest state predominated over the Reward and No-Reward states during wakefulness (Rest vs. Reward: $F_{(1,12)}$=185..1, p<0.001, Rest vs. No-Reward: $F_{(1,12)}$=389.2, p<0.001), while it was progressively less represented from light to deeper sleep stages (main effect of sleep for the Rest state: $F_{(1,12)}$=22.14, p<0.001). Conversely, both game-related brain states did not differ between Reward versus No-Reward states from wakefulness to N2 (Reward vs. No-Reward for Wake: $F_{(1,12)}$=2.1, p=0.18, N1: $F_{(1,12)}$=0.002, p=0.96, N2: $F_{(1,12)}$=0.22, p=0.64). By contrast, and as demonstrated in the original analysis, the Reward state differed from the No-Reward state during N3 sleep ($F_{(1,12)}$=15.65, p=0.002, Supplementary Fig. 5B).

We also extracted the power values for the relevant frequency bands from the EEG data over the whole sleep session, and correlated these values with the time course of likelihood for the 3 different states detected in the fMRI data from each participant. The resulting correlation values entered an ANOVA with Brain state (Reward, No-Reward, Rest) and Frequency band (low delta, 1-2 Hz; high delta, 2-4 Hz; theta, 4-7 Hz; alpha, 8-10 Hz; sigma, 12-14 Hz; beta, 15-25 Hz) as within-subjects factor. We observed a main effect of Brain state ($F_{(2,24)}$=7.35, p=0.003), a main effect of Frequency band ($F_{(5,60)}$=9.19, p<0.001), and an interaction between both factors ($F_{(10,120)}$=14.77, p<0.001). More specifically, a direct comparison between Reward and No-Reward states (planned comparison) showed a significant difference ($F_{(1,12)}$=5.84, p=0.032) due to the strong positive correlation between low delta activity and the strength of the reactivation of the brain state associated with the successful game (Supplementary Fig. 5C). No such interaction was found when exploring correlations of game-related states with other frequency bands.

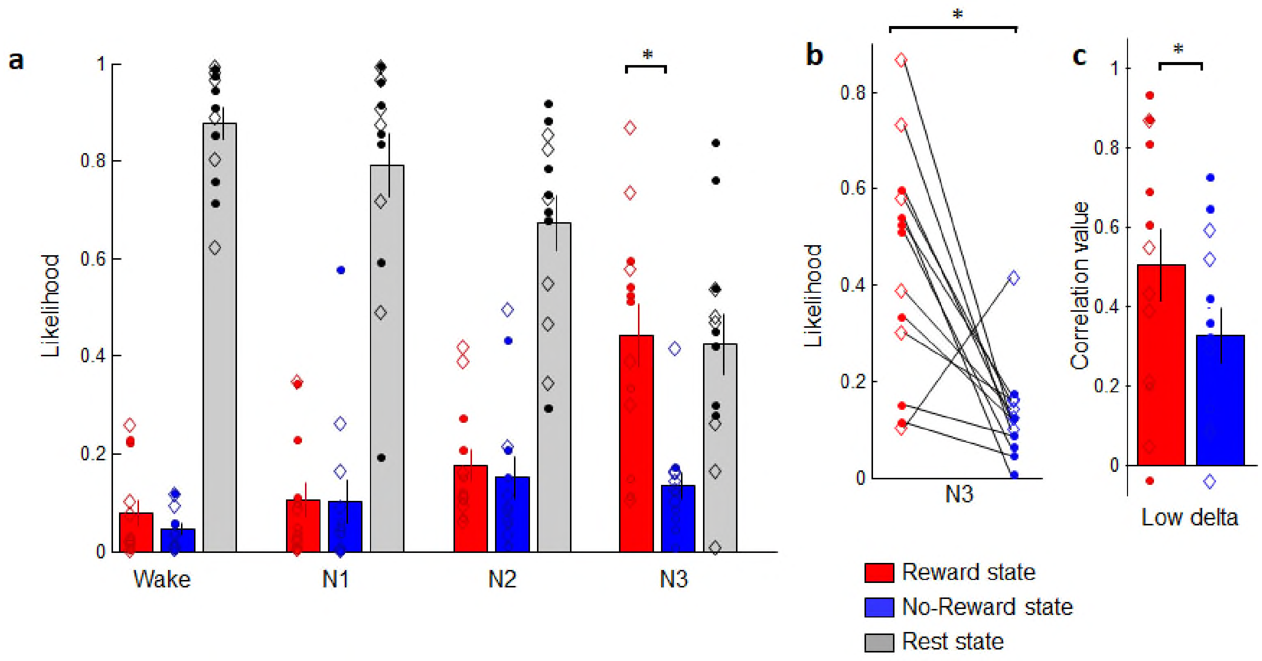

<u>Supplementary Figure 5.</u> Classification results with only 3 states (N=13). (A) Mean likelihood of Reward, No-Reward, Rest brain states for each sleep stage, showing increased reactivation of the brain state associated with the rewarded game during N3. (B) Individual data for the Reward and No-Reward state during N3 sleep. (C) Correlation between time course of likelihood and low delta power (1-2 Hz) for the Reward and No-Reward states. Error bars represent SEM (Standard Error of the Mean); dots represent individual data points.

Next, we ran the fMRI analysis with likelihood values for the task-specific brain states (face, maze) for each fMRI volume during N3 sleep as regressors in a whole-brain regression analysis, independently of the reward status and computed a conjunction analysis, as described in the previous version of the manuscript. We observed a similar set of brain regions activated as when using 5 brain states in the classification (see Figure 4). We also extracted the beta values from each network and correlated the mean beta values with memory performance (Supplementary Fig 6B). Significant correlations were obtained between memory performance and fMRI signal during N3, thus replicating the original results.

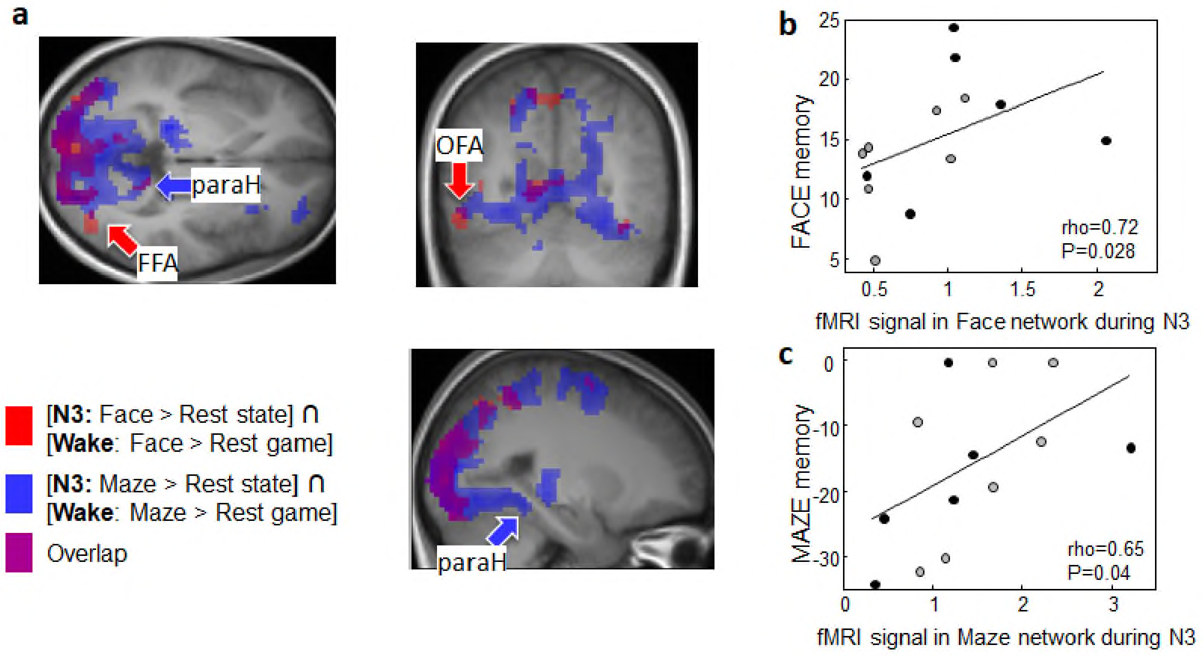

<u>Supplementary Figure 6.</u> Game-specific reactivation during N3 and correlation with subsequent memory using 3 game-related states (N=13). (A) In red, brain regions more activated for Face than Rest brain state during N3 sleep, and also recruited during the execution of the task at wake (blocks of Face vs. Rest; conjunction analysis). In blue, brain regions more activated for Maze than Rest brain state during N3 sleep, and also recruited during the maze game. In purple, overlap between those face and maze reactivation networks during sleep. (B) Correlation between activity in the face network (red regions in A) during N3 and memory performance for the face game. (C) Correlation between activity of the maze network (blue regions in A) during N3 and memory for the maze game. Grey dots for participants who won the face game; black dots for participants who won the maze game. FFA: fusiform face area; paraH: parahippocampus; OFA: occipital face area. N=13.

Finally, we assessed the correlation between the time courses of activity from bilateral hippocampus and bilateral VTA and the 3 different brain states decoded during the sleep session (Reward, NoReward and Rest), and performed two ANOVAs on the rho values, with the 3 states as a within-subjects factor (Supplementary Fig. 7). For the hippocampus, we observed a main effect of State ($F_{(2.24)}$=98.1, p<0.001). Post-hoc analyses revealed that the time course of hippocampal activity correlated significantly more with the Reward state than with the other states (vs. No-Reward state: $F_{(1,12)}$=12.8, p=0.004; vs. Rest state: $F_{(1,12)}$=100.9, p<0.001). Similarly, for the VTA, we observed a main effect of State ($F_{(2.24)}$=25.1, p<0.001), due to the time course of VTA activity correlating more with the Reward state than the other ones (vs. No-Reward state: $F_{(1,12)}$=10.2, p=0.007; vs. Rest state: $F_{(1,12)}$=28.3, p<0.001).

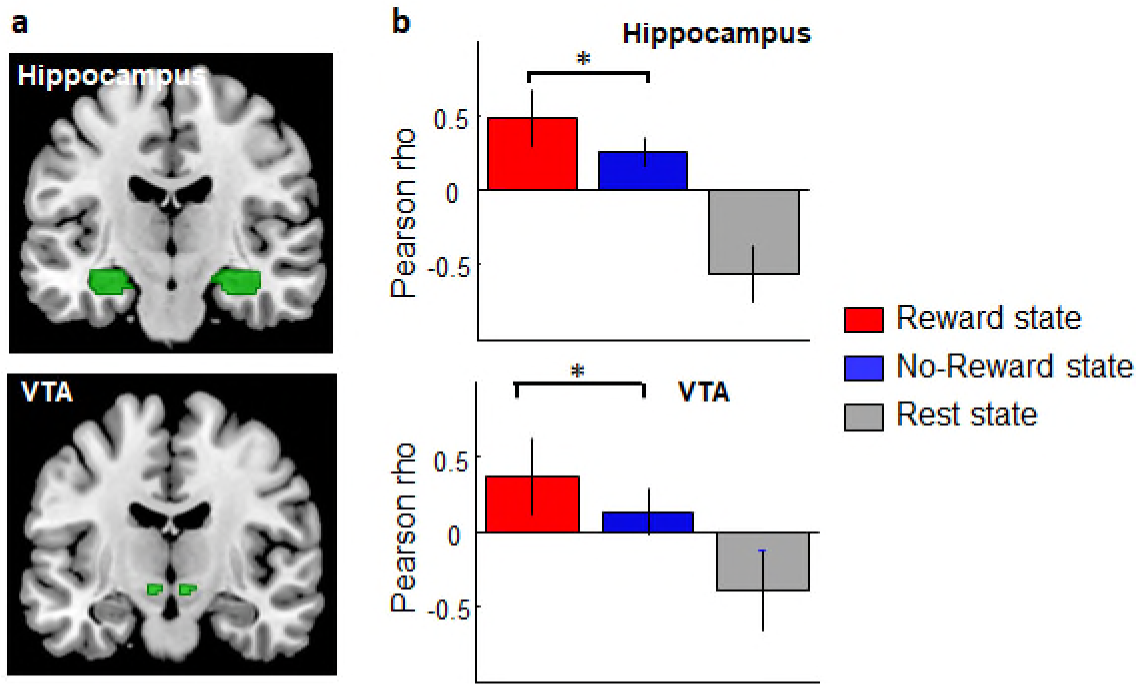

Supplementary Figure 7. Reward-related reactivation in hippocampal and ventral tegmental area (VTA) during sleep, computed for the results of the classifier on only 3 game-related states (N=13). (A) Anatomical masks used as regions of interest (in green). (B) Mean correlation values for all participants between the time courses of the regions of interest and the different brain states. Asterisks indicate P<0.01 for the Reward vs. No-Reward comparison Error bars represent SD (Standard Deviation).

In sum, the confusion matrix demonstrated that, despite the fact that the pre-Face and pre-Maze are of shorter duration and lower accuracy as compared to the other states, these periods are useful for the categorization of brain states during sleep. Theoretically, adding pre-game conditions could either improve or decrease the efficiency of the classifier. The additional analyses demonstrate that the pre-game periods actually accounted for some additional variance in the data and that the main results remain similar with and without these 2 states.
