# Supplementary material for: Reward biases spontaneous neural reactivation during sleep
Source: Nat Commun. 2021 Jul 6;12:4162. doi: 10.1038/s41467-021-24357-5 (PMC8260738; doi:10.1038/s41467-021-24357-5)
Supplement: Supplementary file 3 — Reporting summary [file 41467_2021_24357_MOESM3_ESM.pdf]

nature research

Corresponding author(s): Virginie Sterpenich

Last updated by author(s): Jan 15, 2021

Reporting Summary

Nature Research wishes to improve the reproducibility of the work that we publish. This form provides structure for consistency and transparency in reporting. For further information on Nature Research policies, see our Editorial Policies and the Editorial Policy Checklist.

Statistics

For all statistical analyses, confirm that the following items are present in the figure legend, table legend, main text, or Methods section.

n/a	Confirmed	
☐	☒	The exact sample size (*n*) for each experimental group/condition, given as a discrete number and unit of measurement
☐	☒	A statement on whether measurements were taken from distinct samples or whether the same sample was measured repeatedly
☐	☒	The statistical test(s) used AND whether they are one- or two-sided<br>*Only common tests should be described solely by name; describe more complex techniques in the Methods section.*
☐	☒	A description of all covariates tested
☐	☒	A description of any assumptions or corrections, such as tests of normality and adjustment for multiple comparisons
☐	☒	A full description of the statistical parameters including central tendency (e.g. means) or other basic estimates (e.g. regression coefficient) AND variation (e.g. standard deviation) or associated estimates of uncertainty (e.g. confidence intervals)
☐	☒	For null hypothesis testing, the test statistic (e.g. *F*, *t*, *r*) with confidence intervals, effect sizes, degrees of freedom and *P* value noted<br>*Give P values as exact values whenever suitable.*
☒	☐	For Bayesian analysis, information on the choice of priors and Markov chain Monte Carlo settings
☒	☐	For hierarchical and complex designs, identification of the appropriate level for tests and full reporting of outcomes
☐	☒	Estimates of effect sizes (e.g. Cohen's *d*, Pearson's *r*), indicating how they were calculated

Our web collection on statistics for biologists contains articles on many of the points above.

Software and code

Policy information about availability of computer code

Data collection	We used the MATLAB toolbox Cogent 2000 (Cogent 2000, http://www.vislab.ucl.ac.uk/cogent_2000.php) for the stimulus presentation and response recording for the face and memory tasks. A modified version of Duke 3D was also used for the maze game. For the generation of the different faces in the face game, we used the FaceGen software (Singular Inversions Inc, Toronto, Canada).
Data analysis	We used SPM8 (Wellcome Department of Imaging Neuroscience, London, UK) for the preprocessing and analysis of the MRI data. We used a custom code for the classifier portion of the fMRI analysis (CRF++: Yet Another CRF toolkit, https://taku910.github.io/crfpp/).<br>We used ActiLife software (ActiGraph, FL, USA) to read the data from the actimeters.<br>The software BrainVision Analyzer 2 (Brain Products GmbH, Gilching, Germany) was used for the preprocessing of the EEG data.

For manuscripts utilizing custom algorithms or software that are central to the research but not yet described in published literature, software must be made available to editors and reviewers. We strongly encourage code deposition in a community repository (e.g. GitHub). See the Nature Research guidelines for submitting code & software for further information.

Data

Policy information about availability of data

All manuscripts must include a data availability statement. This statement should provide the following information, where applicable:

- Accession codes, unique identifiers, or web links for publicly available datasets
- A list of figures that have associated raw data
- A description of any restrictions on data availability

The data sets generated and analyzed during the current study are available on the openneuro.org https://openneuro.org/datasets/ds003574/versions/1.0.2

Field-specific reporting

Please select the one below that is the best fit for your research. If you are not sure, read the appropriate sections before making your selection.

- [x] Life sciences
- [ ] Behavioural & social sciences
- [ ] Ecological, evolutionary & environmental sciences

For a reference copy of the document with all sections, see nature.com/documents/nr-reporting-summary-flat.pdf

Life sciences study design

All studies must disclose on these points even when the disclosure is negative.

Sample size	Because the classifier exploits the multivariate nature of the data in high dimensions, and the model incorporates non-trivial assumptions about data structure, classical estimators become therefore suboptimal in these settings. The large amounts of fMRI data (between 1459 and 3589 scans) available for each and every subject enable useful scenarios for cross-validation, i.e., training the model on part of the data. The assessment of decoding is high and we showed above-chance prediction for variables of interest (76% for the FACE state, 87% for the MAZE state and 93% for the REST state).<br>Moreover, based on a previous similar publications, Haxby et al. (Science 2001) performed a decoding approach on similar face and place items. Data from 6 subjects with a mean of 1036 fMRI scans led to an identification accuracy of 94% ±7% for faces and 99% ± 1% for places. Horikawa et al. (Science 2013) used a neural decoding approach on EEG-fRMI data while participants were sleeping, as in our experiment. Three peoples participated in this study, with 266-307 sessions of interest and authors reported a mean decoding accuracy of 60.0%, 95% confidence interval (CI). Finally, Rasch et al. (Science 2007) acquired EEG/fMRI data while 12 participants were sleeping and observed significant activation of the hippocampus. Thus, the sample size for the decoding procedure (18 volunteers for training the model, then applied to 13 participants during deep sleep) is therefore in accordance with previous literature with similar protocols.
Data exclusions	The data from 8 participants were removed from further analysis because of technical problems during scanning (N=4), because the participants did not win at any game (N=2), or because they did not sleep during the sleep session (N=2). The fMRI data from 18 participants were thus used for the analysis of the game session and classifier training (12 women; mean age ± SD: 22.1 ±2.4). Then, the trained classifier was applied to the fMRI data from the sleep session. Because we expected neural reactivation to predominate during periods of sleep with high amounts of slow oscillations, we analyzed the results from those 13 participants who had sustained N3 in the scanner (9 women; mean age ± SD: 22.0 ± 2.5). The exclusion criteria about winning at any game and about sleep depth were pre-established.
Replication	We replicated the decoding approach using 3 states instead of 5 states, demonstrating the robustness of the main results. We also showed that the increased reactivation of the rewarded game was valid when slitting the sample into two independent groups, separating participants who won the face game from those who won the maze game.
Randomization	Participants were allocated to the FACE and MAZE win groups according to their performance during the last 2 blocks of the game session, during which they could potentially win at either game (thanks to useful clues). As soon as one of the games was won, no further informative clues were provided for the unsuccessful game so as to avoid winning at both games. Note that 2 participants were excluded from further analysis because they did not win at any of the games (see Data exclusions above).
Blinding	During data acquisition, group assignment was based on each participant's performance at the games, and could not be performed blindly. After data acquisition, all data was labeled using a combination of random letters and strings. Data analysis was conducted in a fully blinded manner, until group differences were analyzed.

Reporting for specific materials, systems and methods

We require information from authors about some types of materials, experimental systems and methods used in many studies. Here, indicate whether each material, system or method listed is relevant to your study. If you are not sure if a list item applies to your research, read the appropriate section before selecting a response.

Materials & experimental systems

n/a	Involved in the study	
x		Antibodies
x		Eukaryotic cell lines
x		Palaeontology and archaeology
x		Animals and other organisms
	x	Human research participants
x		Clinical data
x		Dual use research of concern

Methods

n/a	Involved in the study	
x		ChIP-seq
x		Flow cytometry
	x	MRI-based neuroimaging

Human research participants

Policy information about studies involving human research participants

Population characteristics	Twenty-six right-handed healthy participants (18 women, 8 men, mean age ± SD: 22.0 ± 2.3 years) participated in this study. A semi-structured interview established the absence of neurological, psychiatric, or sleep disorders. All participants were non-smokers, moderate caffeine consumers, and did not take any medication. They were not depressed as assessed by the Beck Depression Inventory (mean ± SD: 1.7 ± 2.0), and had low anxiety levels as assessed by the STAI-T (31.8 ± 5.8). None of the participants suffered from excessive daytime sleepiness as assessed by the Epworth Sleepiness Scale (5.6 ± 3.0) or sleep disturbances as determined by the Pittsburgh Sleep Quality Index Questionnaire (3.1 ± 2.2). Sensitivity to Punishment and Sensitivity to Reward Questionnaire established that none of the participants had extreme sensitivity to reward (37.0 ± 7.7) or punishment(32.5 ± 5.4), nor did they suffer from excessive impulsivity as assessed by the UPPS Impulsive Behavior Scale (90.1 ± 9.6). We also made sure that none of the participants was a regular player of video games.
Recruitment	All participants were recruited through advertisements on university grounds, and selected in accordance to the inclusion criteria. Because advertisements were public, no particular selection biases are expected.
Ethics oversight	This study was approved by the Human Research Ethics Committee from the State of Geneva.

Note that full information on the approval of the study protocol must also be provided in the manuscript.

Magnetic resonance imaging

Experimental design

Design type	Game session: standard block design. Sleep session: resting state, analyzed using a decoding approach and also using continuous covariates such as time course of the game-related brain states identified by the pattern classification, for example (see main text).
Design specifications	Game session: 2 fMRI runs of 20 min each. Each run contained 8 blocks of preparation of the games (3 s), 4 blocks of FACE game (60 s each), 4 blocks of MAZE game (60 s each), and 8 blocks of rest after each game block (each divided into 3 blocks of 30 s). The very last block, corresponding to when one of the game was won, was modeled as a separate condition (regressor).
Behavioral performance measures	During the Game sessions, no measure of performance was computed. We assessed memory for elements from both games two days after the scanning phase, i.e., after one full recovery night.<br>Memory for the face game was tested by asking participants to place each individual face at their original location on the screen. Participants were presented with a grid of 18 empty rectangles corresponding to the locations of the faces during the game. Three points were attributed for the correct location (out of 18 possible locations), 1 point was given for a correct column (out of 6 possible columns), and 0.5 point for a correct row (out of 3 possible rows).<br><br>Memory for the maze game was assessed by placing participants at one specific location in the maze and asking them to find the starting point that was used during the game session as rapidly as possible. Performance was measured as the shortest map distance from the participant's current location after 30 s to the starting (here goal) point.<br><br>We computed z-scores from the face and maze memory tests to be able to compare performance on both memory tasks within the same ANOVA (distances for the maze game were inversed; so that larger z-score indexed better performance).

Acquisition

Imaging type(s)	Functional and Structural
Field strength	3T
Sequence & imaging parameters	Functional images were acquired with a gradient-echo EPI sequence (repetition time [TR]/ echo time [TE]/flip angle = 2100 ms/30 ms/80°) and parallel imaging (GRAPPA; acceleration factor = 2). Each functional image comprised 32 axial slices (thickness = 3.2 mm without gap, FOV = 235 x 235 mm, matrix size = 128 x 84, voxel size: 3.2 x 3.2 x 3.84 mm,) oriented parallel to the inferior edge of the occipital and temporal lobes.<br>The structural image was acquired before the task with a T1-weighted 3D sequence (MPRAGE, TR/inversion time [TI]/ TE/flip angle = 1900 ms/900ms/2.32 ms/ 9°, FOV = 230 x 230 x 173 mm3, matrix size = 256 x 246 x 192 voxels, voxel size = 0.9 mm isotropic).
Area of acquisition	Whole brain coverage

Diffusion MRI ☐ Used ☒ Not used

Preprocessing

Preprocessing software	Functional volumes were analyzed by using Statistical Parametric Mapping 8 (SPM8; www.fil.ion.ucl.ac.uk/spm/software/

	spm8) implemented in Matlab. Functional MRI data were realigned to the mean image and corrected for head motion and slice timing. Images were then spatially normalized and spatially smoothed with a Gaussian kernel of 8 mm full width at half maximum (FWHM).
Normalization	Functional images were spatially normalized to an echo planar imaging template conforming to the Montreal Neurological Institute (MNI) template (voxel size, 3 × 3 × 3 mm). Parameters were computed from the mean to the template and these parameters were then applied on the functional images.<br>The structural images were normalized on the MNI T1 template .
Normalization template	Standard MNI templates from SPM8
Noise and artifact removal	none
Volume censoring	none

Statistical modeling & inference

Model type and settings	Game session: we used a General Linear Model (GLM) approach conducted in two subsequent steps, accounting for intra-individual (fixed effects) and inter-individual (random effects) variance. For each participant, brain responses at every voxel were fitted with a GLM, and main contrasts of interest were computed. Movement parameters estimated during realignment were added as regressors of no interest in the first-level analyses. The resulting individual maps of t-statistics were then used in second-level random-effects analyses. We used one-sample t-tests for testing common effects in both FACE-win and MAZE-win groups and to identify regions of interest for the decoding approach. Specifically, we selected peaks of activation from clusters of >50 voxels (p<0.001 uncorrected) and as well as peaks in smaller clusters from well-documented task-related regions (i.e. FFA and OFA for the face game).<br>We also used the time course of the game-related brain states identified by the pattern classification (FACE, MAZE and REST) as regressors in an SPM design matrix that we convolved with the hemodynamic function. Again, we computed main contrasts of interest at the first level and we used the contrast maps of each subject at the second level with a one-sample t-test.<br>Decoding approach: we used a classifier based on Conditional Random Fields (CRFs), whose predictions can account for temporal context, such as the different event episodes in our fMRI task paradigm. We performed 4 different steps: 1) definition of the states and ROIs based on the fMRI data from the Game session, 2) training of a classifier on the fMRI data of the Game session at wake extracted from the ROIs (We extracted the time-course of fMRI activity from spheres (radius of 5 mm) centered on the peak of activation for each ROI), 3) validation of the classifier using a leave-one-out procedure, 4) application of the classifier to the Sleep fMRI data, so as to get the likelihood for each brain state to be present in each brain volume acquired during the Sleep session.
Effect(s) tested	Game session: contrasts of interest were FACE > MAZE, MAZE > FACE, FACE-rest1 < FACE-rest3 and MAZE-rest1 < MAZE-rest3.<br>Sleep session: contrasts of interest were FACE state > REST state and MAZE state > REST state.

Specify type of analysis: ☐ Whole brain ☐ ROI-based ☒ Both

Anatomical location(s)	The 58 functional ROIs used for the decoding approach corresponded to brain regions significantly activated during the Game session, as identified by whole-brain contrasts (see above for contrasts used).<br>We created one anatomical mask for the bilateral hippocampus using the AAL atlas and another anatomical mask for the bilateral VTA that we delineated on proton-density images from an independent sample of 19 young males, healthy participants (mean ± SD: 22.05 ± 2.78 years old).

Statistic type for inference (See Eklund et al. 2016)	Statistical inferences were corrected for multiple comparisons according to the Gaussian random field theory using small volumes created from anatomical regions of the AAL atlas.
Correction	FWE

Models & analysis

n/a	Involved in the study
☒	☐ Functional and/or effective connectivity
☒	☐ Graph analysis
☒	☐ Multivariate modeling or predictive analysis
